# Mycetoma and Chromoblastomycosis: Perspective for Diagnosis Improvement Using Biomarkers

**DOI:** 10.3390/molecules25112594

**Published:** 2020-06-02

**Authors:** Anne-Lise Bienvenu, Stephane Picot

**Affiliations:** 1 Service Pharmacie, Groupement Hospitalier Nord, Hospices Civils de Lyon, 69004 Lyon, France; 2 Service d’Hématologie, Groupement Hospitalier Nord, Hospices Civils de Lyon, 69004 Lyon, France; 3 Malaria Research Unit, University Lyon, ICBMS, UMR 5246 CNRS INSA CPE, Campus Lyon-Tech La Doua, F-69100 Lyon, France; stephane.picot@univ-lyon1.fr; 4 Groupement Hospitalier Nord, Institut de Parasitologie et Mycologie Médicale, Hospices Civils de Lyon, 69004 Lyon, France

**Keywords:** biomarkers, mycetoma, chromoblastomycosis, neglected tropical disease, diagnosis

## Abstract

Background: Mycetoma and chromoblastomycosis are both chronic subcutaneous infectious diseases that pose an obstacle to socioeconomic development. Besides the therapeutic issue, the diagnosis of most neglected tropical diseases (NTD) is challenging. Confirmation using direct microscopy and culture, recognized as WHO essential diagnostic tests, are limited to specialized facilities. In this context, there is a need for simple user-friendly diagnostic tests to be used in endemic villages. Methods: This review discuss the available biomarkers that could help to improve the diagnostic capacity for mycetoma and chromoblastomycosis in a theoretical and practical perspective. Results: A lack of research in this area has to be deplored, mainly for mycetoma. Biomarkers based on the immune response (pattern of leucocytes, antibody detection), the dermal involvement (extracellular matrix monitoring, protein expression), and the presence of the infectious agent (protein detection) are potential candidates for the detection or follow-up of infection. Conclusion: Confirmatory diagnosis based on specific diagnostic biomarkers will be the basis for the optimal treatment of mycetoma and chromoblastomycosis. It will be part of the global management of NTDs under the umbrella of stewardship activities.

## 1. Introduction

Mycetoma and chromoblastomycosis are both chronic subcutaneous infectious diseases that pose a devastating obstacle to public health, poverty reduction, and socioeconomic development. For these reasons, they were formally recognized by the World Health Organization (WHO) as neglected tropical diseases (NTD) in 2016. Mycetoma is caused by different species of fungi (eumycetoma) or aerobic filamentous bacteria (actinomycetoma) [1], whereas chromoblastomycosis is caused only by fungi. The causative organisms of eumycetoma including *Madurella mycetomatis*, *Trematosphaeria grisea*, and *Scedosporium apiospermum*, are distributed worldwide, but the main endemic areas, known as the Mycetoma Belt, include the Bolivarian Republic of Venezuela, Chad, Ethiopia, India, Mauritania, Mexico, Senegal, Somalia, Sudan, and Yemen [2]. The clinical characteristics of mycetoma include local swelling, multiple sinuses, and discharge which contains the infective forms, known as “grains”. Mycetoma usually affects the foot, leading to substantial disability in advanced cases due to bone destruction by the infectious agent, and in some cases, may be fatal following secondary bacterial septicemia. Indeed, many patients are late presenters with advanced infection, given the painlessness of the disease and the scarcity of medical and health facilities in endemic areas, for whom amputation may be the only available treatment. The causative fungi of chromoblastomycosis, including *Fonsecaea pedrosoi* and *Cladophialophora carrionii*, are distributed worldwide, but the highest prevalence of the disease is found in the Amazon region of Brazil, the northern part of Venezuela, Costa Rica, the Dominican Republic, and in Madagascar [2]. Clinical presentations of chromoblastomycosis are polymorphic, the most frequent being nodular, verrucous, and tumoral-like. Mycetoma and chromoblastomycosis are treated using intensified disease management, including anti-infective agents (antibiotic or antifungal) and/or surgical treatment based on the needs and clinical expression of disease in each individual patient [3]. However, most of the current medicines have limited effectiveness, many side effects, and are not available in endemic countries because of their high costs. Besides the therapeutic issue, the diagnosis of NTDs is challenging. It was reported in a recent expert consensus report that in basic healthcare settings, direct microscopy combined with clinical signs were the most useful diagnostic indicators to prompt referral for treatment [4]. Moreover, microscopy and culture are now recognized as WHO essential diagnostic tests. However, in endemic countries with limited health access, the initial visual recognition from the clinical examination of a suspected case may be unavailable. Confirmation using direct microscopy and culture are limited to specialized facilities. In this context, there is a need of simple user-friendly diagnostic tests to use in mycetoma- and chromoblastomycosis-endemic villages. The aim of this review is to discuss the available biomarkers that could help to improve the diagnostic capacity for mycetoma and chromoblastomycosis and to discuss innovative diagnostic solutions in a theoretical and practical perspective.

## 2. Mycological Diagnosis of Mycetoma and Chromoblastomycosis

The diagnosis of mycetoma and chromoblastomycosis requires laboratory confirmation by direct examination and/or histopathology. Mycetoma direct examination is based on the morphological and physiological characteristics of the grains discharged from sinuses, whereas the observation of muriform cells in clinical specimens is compulsory for the diagnosis of chromoblastomycosis. Although direct examination is helpful in detecting the diseases, it is important to culture the causative organism properly. Indeed, some species may be resistant to antifungals. In addition, identification may contribute to data on the epidemiology and biodiversity of the etiological agents worldwide. The common methods used for the identification of pathogenic fungi isolated from culture are based on the microscopic examination of morphological characteristics allowing identification to the genus level. However, these methods are time-consuming, require expertise in microscopy, and have low specificity. 

Further identification to the species level requires molecular approaches based on PCR amplification and the sequencing of conserved genomic regions. Molecular tools are especially needed for the implementation of treatment and/or disease surveillance. For instance, the agents of the disease may be morphologically indistinguishable from their environmental counterparts, or cryptic species such as *F. pedrosoi*, *F. monophora*, and *F. nubica*, may coexist, but with different virulence and invasive potentials. As an example, new molecular tools to distinguish *Fonsecaea* involved in chromoblastomycosis have been developed based on padlock probes in rolling circle amplification; these tools allow the detection and species differentiation of *Fonsecaea* agents without sequencing [5]. Molecular approaches are based on PCR amplification and the sequencing of conserved genomic regions and have been shown to allow the resolution of the causative agents to the species level, but these approaches are onerous and relatively time-consuming. Besides molecular-based identification, matrix-assisted laser desorption ionization–time of flight (MALDI-TOF) mass spectrometry (MS) has been shown to provide a robust, cost-effective, and rapid identification at the species level of a variety of fungi from pure culture. As an example, the identification of the agents of black-grain mycetoma by MALDI-TOF-MS demonstrated the accurate identification of eumycetoma agents and related fungi [6]. 

Despite the recognition of microscopy and culture as WHO essential diagnostic tests, these tests are still limited to specialized facilities in endemic areas. The identification of fungi at the species level, while useful to optimize disease management, is also not available in most endemic areas.

## 3. Pattern of Leukocytes and Cytokines

Considering the inflammatory nature of the diseases, the pattern of leucocytes is one opportunity to detect the infection. This strategy was reported for both mycetoma and chromoblastomycosis. 

During mycetoma, suppurative granulomas (composed of neutrophils) surrounding characteristic grains were described in the subcutaneous tissue [7]. The neutrophilic infiltrate is surrounded by histiocytes and a mixed inflammatory infiltrate, including lymphocytes, plasma cells, eosinophils, and macrophages. Skin lesions of actinomycetoma (ACM) and eumycetoma (EUM) were studied to compare cell elements in the inflammatory infiltrate [8]. In both groups of mycetoma, CD4 and CD8 T lymphocytes were identified surrounding the neutrophil aggregates with macrophages, whereas B lymphocytes were not identified. Interestingly, a higher number of CD8+/lymphocytes (p = 0.02) and macrophages (p = 0.01) were observed in ACM lesions compared to EUM lesions [8]. The pattern of leukocytes may be a valuable approach to distinguish ACM and EUM that would benefit from different anti-infective treatments. 

The histology of chromoblastomycosis is characterized by a foreign body organized granuloma with isolated areas of microabscess formation mainly composed of giant cells and groups of fungal cells. It was reported that CD4+ and CD8+ T lymphocyte populations, B lymphocytes, neutrophils, and macrophages play a significant role in the cell-mediated response during chromoblastomycosis [9]. Populations of macrophages, lymphocytes, neutrophils, and Langerhans cells and their correlation with the expression of macrophage inflammatory protein-1α (MIP-1α), chemokine receptors (CXCR3, CCR1), and enzymes (superoxide dismutase, SOD, and nitric oxide synthase, iNOS) were indeed studied in order to better characterize the cell-mediated immune reactivity of chromoblastomycosis [10]. Biopsies of patients with a clinical and histopathological diagnosis of chromoblastomycosis were studied using immunohistochemistry before the beginning of treatment. Fungi numbers were correlated with CD3, CD45RO, and iNOS positive cells. Furthermore, MIP-1α expression was associated with CD45RO, CD68, iNOS, and CXCR3. The authors suggested the possible role of MIP-1α in the fungi persistence during chromoblastomycosis and the regulatory role of macrophage activation in determining the outcome and fungal destruction in chromoblastomycosis infections. These results suggested the potential use of MIP-1α as a prognostic biomarker. In another study [11], it was demonstrated that monocytes from patients with different clinical forms of chromoblastomycosis had distinct phenotypic and functional profiles. A higher production of IL-10 and a lower expression of HLA-DR and costimulatory molecules was indeed produced by monocytes of patients presenting a more severe form of the disease. This was confirmed by Mazo Fávero Gimenes et al., who demonstrated a predominant production of IL-10 with the inhibition of IFN-ƴ in patients with more severe clinical forms, resulting in the low-level induction of T cells compared to patients with mild chromoblastomycosis; mild forms of CBM favor a Th1 profile that may inhibit disease development, while moderate forms trigger an intermediate response between Th1 and Th2 [12]. The monocyte subsets, as well as the Th1/Th2 profile, may be valuable in identifying patients with severe forms of chromoblastomycosis and may be used as a prognostic biomarker.

This approach, based on the pattern of leukocyte subpopulations, is not specific enough to be used as a diagnostic tool. As stated by the authors, it may be used at the time of diagnosis to distinguish the two mycetoma entities or as a prognostic tool during chromoblastomycosis. 

## 4. Antibodies

Antibody production may be observed during mycetoma or chromoblastomycosis as a consequence of the host response to the presence of an infectious agent. Antibodies can be directed towards the infectious agent itself or toward immune cells of the host in relation with the infection. This was observed during chromoblastomycosis, leading to the secretion of anti-*Fonsecaea pedrosoi* IgG, as well as anti-neutrophil cytoplasmic antibodies (ANCAs).

### 4.1. Anti-F. pedrosoi IgG

In patients with severe chromoblastomycosis, the level of anti- *F. pedrosoi* IgG (IgG1, IgG2, and IgG3) was higher compared to patients with moderate or mild disease (*p* < 0.05) [13]. After treatment, the mean antibody titers of IgG, IgG1, and IgG2 were reduced (*p* < 0.05) [14]. Furthermore, a reduction in IgG3 and IgG titers was observed in patients with a rapid response (*p* < 0.05) and a reduction in IgG2 in patients with rapid and intermediate responses (*p* < 0.05) [13]. Interestingly, the immunological analysis showed that the antibody anti-*F. pedrosoi* did not provide protection against infection [13]. The detection of anti- *F. pedrosoi* IgG may provide a specific diagnostic biomarker of chromoblastomycosis caused by *F. pedrosoi*.

### 4.2. Anti-Neutrophil Cytoplasmic Antibodies (ANCAs)

Patients suffering chromoblastomycosis were tested for the presence of ANCAs [14]. Among them, 20% had detectable ANCAs. This study demonstrates that chromoblastomycosis triggers autoreactivity against myeloid lysosomal antigens. ANCAs may have a place in the diagnosis of chromoblastomycosis, whereas this biomarker will not be specific to the infection.

## 5. Monitoring of the Extracellular Matrix 

During chromoblastomycosis, there is a marked pseudoepitheliomatous hyperplasia of the epidermis, and in some areas, the apparent transepidermal elimination of fungal cells, which can be found in the stratum corneum [15]. The subcutaneous pathology of chromoblastomycosis led to the study of the extracellular matrix during this infection, looking at potential metabolites that could help the diagnosis. The collagen content and the turn-over of the extracellular matrix, based on serum and urinary metabolites (pyridinoline and pentosidine), were then monitored in patients with a diagnosis of chromoblastomycosis [16]. The serum level of type III collagen was correlated with the lesion size. In patients whose lesion size reduced by more than 50% during terbinafine treatment, urinary pyridinoline was higher compared to patients whose lesion size did not significantly reduce. It was demonstrated that pyridinoline and pentosidine cross-links increased in the lesions during treatment, whereas a significant reduction in collagen content was observed [16]. The monitoring of collagen content and cross-linking in chromoblastomycosis patients could be used as a biomarker for diagnosis and treatment follow-up. Whereas the monitoring of extracellular matrix was not studied during mycetoma, it would be worth evaluating this aspect of the disease, considering that mycetoma is clinically characterized by a subcutaneous mass. In any case, this biomarker will not be specific to the disease, but more useful as a biomarker of treatment efficacy.

## 6. Protein Expression

The presence of an infectious agent may lead to protein secretion in relation with the infectious agent itself or as a consequence of its presence in the host, leading to an epidermal proliferation.

### 6.1. Translationally Controlled Tumor Protein 

In the case of mycetoma, a protein homologous to the translationally controlled tumor protein (TCTP) was demonstrated to be present in *M. mycetomatis* [17]. Indeed, TCTP was secreted into the culture medium and was expressed on hyphae present in the black grains of eumycetoma. Moreover, significant IgG and IgM immune responses against TCTP were demonstrated. Interestingly, the antibodies’ levels correlated with lesion size and disease duration, as demonstrated by the highest levels of antibodies after a disease duration of 6–15 years. TCTP is the first well-characterized immunogenic antigenic for the fungus *M. mycetomatis*. The authors concluded that TCTP is the first monomolecular vaccine candidate [17]. Another perspective could be the use of TCTP as a diagnostic and prognostic biomarker, and this will be specific to mycetoma, as TCTP was proven to be secreted by the infectious agent.

### 6.2. Galectin-3 Expression 

During chromoblastomycosis, known as a benign epidermal proliferation, it was suggested that galectin-3 may be an interesting biomarker. Galectin-3 is indeed expressed in basal cell carcinoma and squamous cell carcinoma, in relation with tumor genesis, progression, and metastasis. Galectin-3 expression was studied by immunohistochemistry on skin sections of patients suffering chromoblastomycosis [18]. A significant downregulation of galectin-3, both in selected benign skin diseases and in skin cancers, was demonstrated. This result indicates that a regulatory pathway of galectin-3 expression during epidermal hyperplasia occurs independently of the differentiation status of keratinocytes [18]. Then, galectin-3 expression may be used as a diagnostic biomarker of chromoblastomycosis. This biomarker should be studied during mycetoma, considering that mycetoma is also an epidermal hyperplasia, whereas this will not be specific to this infectious disease.

## 7. Conclusions

Considering that mycetoma and chromoblastomycosis are inflammatory subcutaneous infectious diseases, biomarkers based on the immune response (pattern of leucocytes, antibody detection), the dermal involvement (extracellular matrix monitoring, protein expression), and the presence of the infectious agent (protein detection) are potential candidates for the detection or follow-up of infection. A lack of research in this area has to be deplored mainly for mycetoma, despite the urgent need for such biomarkers. Advances in identifying specific diagnostic biomarkers of mycetoma and chromoblastomycosis may indeed pave the way for new laboratory-based or point-of-care tests. Most of the biomarkers described in this review are indeed not specific or applicable as prognostic biomarkers, which would be required subsequently. Confirmatory diagnosis will be the basis for the optimal treatment of mycetoma and chromoblastomycosis. It will be part of the global management of NTDs under the umbrella of stewardship activities.
